# Modeling solubility of CO_2_–N_2_ gas mixtures in aqueous electrolyte systems using artificial intelligence techniques and equations of state

**DOI:** 10.1038/s41598-022-07393-z

**Published:** 2022-03-07

**Authors:** Reza Nakhaei-Kohani, Ehsan Taslimi-Renani, Fahime Hadavimoghaddam, Mohammad-Reza Mohammadi, Abdolhossein Hemmati-Sarapardeh

**Affiliations:** 1 Department of Chemical & Petroleum Engineering, Shiraz University, Shiraz, Iran; 2 Department of Electrical Engineering, University of Malaya (UM), 50603 Kuala Lumpur, Malaysia; 3 Ufa State Petroleum Technological University, Ufa, 450064 Russia; 4 Institute of Unconventional Oil & Gas, Northeast Petroleum University, Heilongjiang, Daqing, 163318 China; 5 Department of Petroleum Engineering, Shahid Bahonar University of Kerman, Kerman, Iran; 6 Key Laboratory of Continental Shale Hydrocarbon Accumulation and Efficient Development, Ministry of Education, Northeast Petroleum University, Daqing, 163318 China

**Keywords:** Energy science and technology, Engineering

## Abstract

Determining the solubility of non-hydrocarbon gases such as carbon dioxide (CO_2_) and nitrogen (N_2_) in water and brine is one of the most controversial challenges in the oil and chemical industries. Although many researches have been conducted on solubility of gases in brine and water, very few researches investigated the solubility of power plant flue gases (CO_2_–N_2_ mixtures) in aqueous solutions. In this study, using six intelligent models, including Random Forest, Decision Tree (DT), Gradient Boosting-Decision Tree (GB-DT), Adaptive Boosting-Decision Tree (AdaBoost-DT), Adaptive Boosting-Support Vector Regression (AdaBoost-SVR), and Gradient Boosting-Support Vector Regression (GB-SVR), the solubility of CO_2_–N_2_ mixtures in water and brine solutions was predicted, and the results were compared with four equations of state (EOSs), including Peng–Robinson (PR), Soave–Redlich–Kwong (SRK), Valderrama–Patel–Teja (VPT), and Perturbed-Chain Statistical Associating Fluid Theory (PC-SAFT). The results indicate that the Random Forest model with an average absolute percent relative error (AAPRE) value of 2.8% has the best predictions. The GB-SVR and DT models also have good precision with AAPRE values of 6.43% and 7.41%, respectively. For solubility of CO_2_ present in gaseous mixtures in aqueous systems, the PC-SAFT model, and for solubility of N_2_, the VPT EOS had the best results among the EOSs. Also, the sensitivity analysis of input parameters showed that increasing the mole percent of CO_2_ in gaseous phase, temperature, pressure, and decreasing the ionic strength increase the solubility of CO_2_–N_2_ mixture in water and brine solutions. Another significant issue is that increasing the salinity of brine also has a subtractive effect on the solubility of CO_2_–N_2_ mixture. Finally, the Leverage method proved that the actual data are of excellent quality and the Random Forest approach is quite reliable for determining the solubility of the CO_2_–N_2_ gas mixtures in aqueous systems.

## Introduction

In the last decade, one of the most important challenges in the petroleum and chemical industries has been evaluating the solubility of different gases in liquids, including hydrocarbon and non-hydrocarbon gases^1–3^. The solubility of gases in liquids can be vital in the petroleum and chemical industries for a variety of reasons, including transport operations and the production of hydrates^1,4^. CO_2_ as a greenhouse gas has been considered a serious problem in recent decades^5–7^. Carbon capture and storage (CCS)^8,9^ is a technique that involves capturing CO_2_ from major point sources and storing it in formations^10,11^. Flue gas storage in saline aquifers, as well as CO_2_ extraction and storage using gas hydrates, are considered as potential CCS methods. As a result, information gaps about these methods, such as the solubility of gas mixtures in water and brine, must be filled before commercialization. Due to the high cost of traditional CCS technologies, considerable efforts have been made to improve the efficiency of CCS operations by creating cost-effective and practical CCS approaches; however, there are still a lot of technological and financial roadblocks to overcome^10,12–14^.

Flue gas or the mixture of CO_2_ and N_2_ injected within gas hydrate reservoirs have been suggested as a potential alternative for CO_2_ underground storage. The thermodynamic mechanism by which CO_2_ in flue gas or a CO_2_–N_2_ mixture is collected as hydrate, on the other hand, is not well recognized^15^. CO_2_ storage in hydrate reservoirs has expensive obstacles that limit its widespread usage, despite all of the stated benefits. The primary expense in this scenario is CO_2_ collection before storage^15,16^. Injecting CO_2_–N_2_ mixture within gas hydrate reservoirs rather than pure CO_2_ might considerably cut CO_2_ separation expenses. Furthermore, an industrial-scale CO_2_ substitution experiment on the North Slope of Alaska found that injecting a gas combination of 77/23 ratio of N_2_/CO_2_ into a hydrate reservoir while recovering methane successfully avoided CO_2_ hydrate creation around the injection well. Although the prior studies show that injecting CO_2_–N_2_ gas mixes into gas hydrate reservoirs might be a cost-effective technique for CCS, a primary concern remain: How can the reservoir circumstances following CO_2_–N_2_ mixtures or flue gas injection into a gas hydrate reservoir affect the production of CO_2_ and CO_2_–mixed hydrates^15^? Since different thermodynamic conditions affect the injection process of the CO_2_–N_2_ mixture and make the injection process difficult, the first important step is to evaluate the solubility of the CO_2_–N_2_ mixture at different thermodynamic conditions. It should be noted that these limitations have also led to limited laboratory data on the solubility of CO_2_–N_2_ mixture in liquids. Therefore, finding a solution to measure the solubility of the CO_2_–N_2_ mixture has great importance. As a result of these considerations, assessing the solubility of gases in liquids has become a contentious issue. CO_2_ and N_2_ have been extensively considered as two frequently used non-hydrocarbon gases in recent studies^17,18^. The injection of CO_2_ into the aquifer and the injection of a mixture of CO_2_ and N_2_ into oil and gas reservoirs are two examples of these situations, where knowing the degree of solubility of the gas is critical^10,19^. As a result, a thorough understanding of the physical and chemical interactions between CO_2_, N_2_, and water is required. For instance, solubility trapping and mineral trapping are the two significant mechanisms that influence the injection of CO_2_ into the aquifer. To accurately determine the effect of these mechanisms, it is necessary to conduct a sufficient number of theoretical and experimental studies, which can be time-consuming and costly^10,20,21^.

In addition to the laboratory experiments, another technique for determining the solubility of CO_2_ and N_2_ in water is to utilize equations of state (EOSs); however, it should be noted that EOSs are more appropriate for pure fluids but have limitations for pure compounds. Some of these limitations are as follows^22,23^:

- To determine the solubility using these types of equations, critical characteristics of pure substances are necessary. Many of the chemicals studied, particularly those with complicated chemical structures, break down before meeting critical conditions. As a result, measuring the relevant characteristics does not appear to be feasible.
- To adjust the thermodynamic coefficients of the equation for a more precise estimation of the physical properties of the system, several physicochemical aspects of the system should be evaluated, such as the characteristics of the donor and the acceptor of the hydrogen bond of the molecule.
- Interaction factors setting for solubility data for each model is a time-consuming process.
- Numerical methods are often divergent to solve some equations for pure materials that have low solubility in water.
- The solubility estimations are heavily influenced by the optimization techniques used to get the best values for the thermodynamic model parameters.

As a result, choosing the appropriate optimization technique is another issue to consider. Despite these flaws, thermodynamic techniques have been extensively used to forecast the solubility of CO_2_, N_2_, and other gases in water, which are often found in the oil and gas industry under a variety of thermodynamic conditions. In the literature, CO_2_ solubility in water and aqueous solutions^24–26^ of salts like NaCl, KCl, and CaCl_2_ has been thoroughly documented. Also, the solubility of N_2_ and CO_2_–N_2_ mixture in water and brine has been studied^22,27–29^. Tomoya et al.^30^ measured CO_2_ solubility in aqueous solutions and then correlated the experimental data with the Peng-Robinson-Stryjek-Vera EOS. Yiteng et al.^31^ also needed to know the solubility of CO_2_ in brine to estimate CO_2_ capturing potential in deep saline aquifers. For this purpose, they utilized the Peng-Robinson Cubic-Plus-Association (PR-CPA) EOS to calculate the solubility of CO_2_ in brine. They represented that good agreement was achieved with laboratory data.

The second group of methods for estimating solubility involves creating correlations, particularly mathematical methods that employ the physical characteristics of the chemicals in a manner that makes these approaches broad and thorough. These techniques may represent/predict the solubility of substances from diverse chemical categories in water in any condition^22^. Abraham et al.^32^ suggested a linear solvation energy relationship (LSER) approach. However, the relationship can predict the solubility of ordinary organic substances; the model's properties are challenging to be determined from the compounds' chemical structures. Other researchers have taken the same method^33,34^.

In the previous studies, a number of experimental data have been reported for the solubility of non-hydrocarbon gases, including CO_2_ and N_2_ in liquids, especially in water^18,35,36^. There is a scarcity of experimental results for non-hydrocarbon solubility due to the difficulties and sophistication of measured data of natural gas including gas equilibrium data. As a result, the utilization of laboratory data in new modeling approaches like artificial neural networks has gotten much attention^1^. Machine learning techniques have recently found widespread use in forecasting challenges such as hydrate formation^37^, ammonia solubility in liquids^38^, simulating asphaltene behavior^39^, and hydrocarbon-CO_2_ interfacial tension^40^. They have received much interest as a result of their captivating performance^41^. Samani et al.^42^ proposed different intelligence techniques for estimating the solubility of various gases in aqueous electrolyte systems. Regarding the solubility of non-hydrocarbon gases (i.e., N_2_ and CO_2_) in aqueous electrolyte systems, their database includes 774 data points, of which only 81 data are related to the N_2_–CO_2_ gas mixture and the rest are related to the solubility of N_2_ and CO_2_ pure gases. Their model was based on Coupled Simulated Annealing (CSA) linked to the Least-Squares Support Vector Machine (LSSVM) method. Average absolute relative error and root mean square error (RMSE) values of their proposed CSA-LSSVM model were 10.71% and 0.0011, respectively. Hemmati-Sarapardeh et al.^43^ investigated the solubility of CO_2_ in water at high pressures and temperatures using four powerful machine learning techniques. In this study, Multilayer Perceptron (MLP), Radial Basis Function (RBF), Least-Squares Support Vector Machine (LSSVM), and Gene Expression Programming (GEP) models were developed using temperature and pressure as input data to estimate the solubility of CO_2_ in water. The results showed that the LSSVM-FFA model a with an RMSE value of 0.3261 had the best performance compared to other models. Nabipour et al.^1^ investigated the solubility of CO_2_ and N_2_ in aqueous solutions using Extreme Learning Machine (ELM) and LSSVM approaches. Their solubility database was similar to Samani et al.'s work^42^ including 774 data points with less than 90 data related to CO_2_–N_2_ mixture solubility. The results showed that the LSSVM technique with an RMSE value of 0.001 had higher proficiency than the ELM approach in estimating the solubility values of CO_2_ and N_2_ in aqueous solutions. Temperature, pressure, and composition were the most critical input parameters to the models. Saghafi et al.^44^ investigated the solubility of CO_2_ in Monoethanolamine (MEA), Diethanolamine (DEA), Triethanolamine (TEA), and N-Methyldiethanolamine (MDEA) aqueous solutions. In this study, the AdaBoost-Decision Tree method and intelligent neural networks were used. The results showed that AdaBoost-Decision Tree models with RMSE values of 0.005–0.022 obtained the best solutions for different aqueous solutions. Gharagheizi et al.^22^ estimated the solubility of pure compounds such as CO_2_ in water using an Artificial Neural Network-Group Contribution (ANN-GC) technique. The results showed that this model with an RMSE value of 0.4 could have a good performance in estimating the solubility of pure materials in water.

Therefore, as mentioned earlier, particular importance and attention to the issue of determining the solubility of CO_2_ and N_2_ in liquids and especially water with various techniques including laboratory methods^45^, EOSs, mathematical methods, and intelligent neural networks^46,47^ in previous studies has caused further studies in this field and is still of interest to researchers. Although many studies have been done on pure CO_2_ and N_2_, few studies investigated the solubility of CO_2_–N_2_ mixtures in water and brine. Only two papers^1,42^ applied intelligent models for CO_2_–N_2_ mixtures, however, they used less than 90 data points and in limited ranges of operating parameters.

In this study, to estimate the solubility of CO_2_–N_2_ mixtures in water and aqueous brine solutions, an extensive database containing 289 laboratory is collected from the literature. This paper uses six machine learning approaches, including Random Forest, Decision Tree (DT), Gradient Boosting-Decision Tree (GB-DT), Adaptive Boosting-Decision Tree (AdaBoost-DT), Adaptive Boosting-Support Vector Machine for Regression (AdaBoost-SVR), and Gradient Boosting-Support Vector Machine for Regression (GB-SVR), for determining CO_2_–N_2_ mixture solubility in aqueous solutions in terms of temperature, pressure, ionic strength of aqueous brine solutions, CO_2_ mole percent in gaseous mixture, and finally the index of non-hydrocarbon gases (i.e., N_2_ and CO_2_) whose solubility is to be estimated. Also, four reputable equations of state, including Peng–Robinson (PR), Soave–Redlich–Kwong (SRK), Valderrama–Patel–Teja (VPT), and Perturbed-Chain Statistical Associating Fluid Theory (PC-SAFT) are utilized to have a comparison with artificial intelligence models. Moreover, the sensitivity analysis of input parameters utilizing the relevancy factor is performed to check their impact on the solubility of CO_2_–N_2_ gas mixtures in aqueous electrolyte systems. Lastly, the Leverage method is applied to investigate the quality of actual data and the reliability of the best-proposed approaches for determining the solubility of the CO_2_–N_2_ gas mixtures in aqueous systems.

## Data gathering

In this study, to estimate the solubility of CO_2_–N_2_ mixtures in water and aqueous brine solutions, an extensive database containing 289 laboratory data has been collected from the literature^10,18^, which is presented in the Supplementary file. Although two studies^1,42^ have been performed to estimate the solubility of CO_2_, N_2_, and CO_2_–N_2_ mixture in aqueous electrolyte systems using artificial intelligence models, in these studies, the number of data related to the solubility of CO_2_–N_2_ mixture in water is much less than the data for the two pure substances (i.e., CO_2_, N_2_). The number of CO_2_–N_2_ mixture solubility data of these studies^1,42^ is less than 90 data points. The database used in this work has 200 data points of CO_2_–N_2_ mixture solubility in aqueous electrolyte solutions more than Nabipour et al.^1^ and Samani et al.'s^42^ works. Therefore, what distinguishes this study from other previous studies is the use of a large data bank containing a large number of data related to CO_2_–N_2_ mixture solubility in aqueous brine solutions. Therefore, the results of the developed models can be more comprehensive and reliable for use in the cases mentioned at the beginning of the introduction. To develop the models, temperature, pressure, ionic strength of aqueous solutions, CO_2_ mole percent in gaseous mixture, and the index of non-hydrocarbon gases (IDX: 1 = N_2_ and 2 = CO_2_) whose solubility is to be estimated, have been used as input parameters. The statistical parameters of inputs and output data are summarized in Table 1.

**Table 1:** Statistical details of the dataset in this work.

	IDX	Temperature (K)	Pressure (MPa)	Ionic strength (M)	CO_2_ (mole %)	Solubility (mole fraction)
Mean	1.505	294.13	11.11	0.8158	31.6114	0.004323
SD	0.5008	15.76	5.83	1.1633	29.1251	0.006132
Min	1	273.25	1.51	0	0	0.0001
Max	2	318.15	21.74	3.99	100	0.025

## Models’ implementation

### Support vector machine for regression (SVR)

The Support Vector Machine (SVM) is a type of controlled machine learning system that can be employed for both regression (SVR) and classification (SVC) problems^48^. SVM has been widely used in various research areas due to its superior feature, notably in solving non-linear problems called the kernel trick, mapping the input space into a higher-dimensional space. For the sake of conciseness, this article briefly explains the concept of SVR; however, it has extensively been presented in literature^49^. Let the given dataset be a set of *n* independent samples, $[\left({x}_{1}, {y}_{1}\right),\dots .,\left({x}_{n}, {y}_{n}\right)]$, where $x\in {R}_{d}$ has d dimension and $y\in R$. The objective of SVR is to identify regression function as below:

1 $$y=f\left(x\right)=w.\phi \left({x}_{i}\right)+b$$

here w, b, and $\phi \left(x\right)$ denote the weight, bias, and kernel function, respectively.

To get the appropriate values of the weight and bias vectors, Vapnik et al.^50^ suggested the following optimization procedure:

$$minimize \frac{1}{2}{w}^{T}w+C\sum_{j=1}^{N}\left({\zeta }_{j}^{-}+{\zeta }_{j}^{+}\right)$$

2 $$\left\{\begin{array}{l}\left(w. \emptyset \left({x}_{i}\right)+b\right)-{y}_{i}\le \varepsilon +{\zeta }_{j}^{-}\\ {y}_{i}-\left(w. \emptyset \left({x}_{i}\right)+b\right)\le \varepsilon +{\zeta }_{j}^{+}\\ {\zeta }_{j}^{+},{\zeta }_{j}^{-}\ge 0 . i=1,2,\dots ,m\end{array}\right.$$

here ${w}^{T}$ indicates the transposed matrix, $\varepsilon$ is the toleration of error, ${\upzeta }_{\mathrm{j}}^{+}$ and ${\upzeta }_{\mathrm{j}}^{-}$ are regarded positive variables reflecting the lower and higher excessive variations, respectively, and *C* interprets a positive regularization factor determining the deviance from $\upvarepsilon$. By employing the Lagrange multiplier, Eq. (2) can be converted into a dual optimization problem as follows, which makes it easier to solve^48^.

3 $$y=f\left(x\right)=\sum_{i=1}^{n}\left({a}_{i}-{a}_{i}^{*}\right).K\left({x}_{i}, \mathrm{x}\right)+b$$

where $K({x}_{k},{x}_{l})$ is the kernel function, ${a}_{k}$ and ${a}_{k}^{*}$ represent the Lagrange multipliers.

It should be noted that in the present study, the polynomial kernel function was used in the SVR model which was selected by using grid search for the best performance. Weight and bias in Eq. (1) stand for trainable variables of SVR model.

### Random forest (RF)

**Figure 1:**
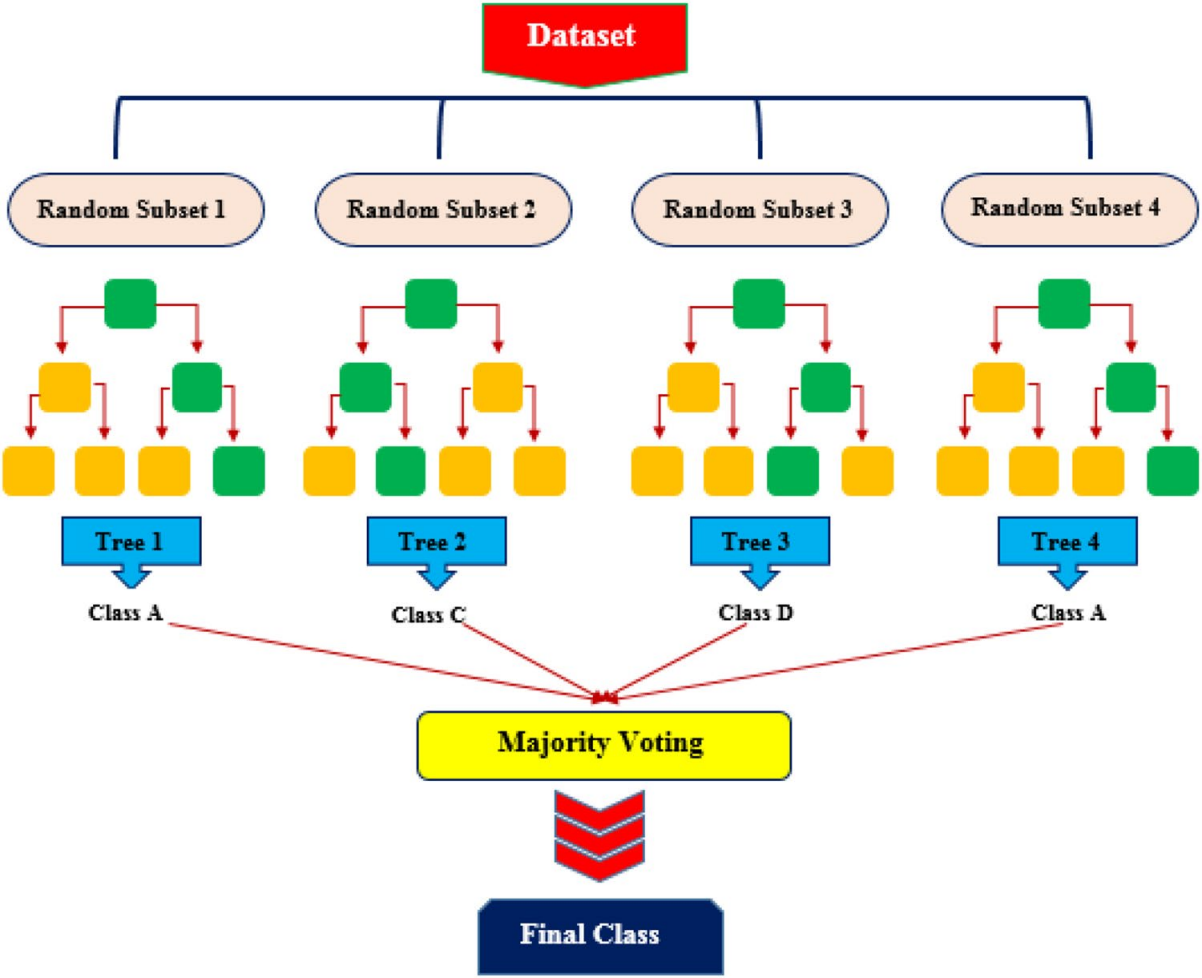
Schematic illustration of random forest algorithm.

Decision Trees, a tree-like structure, are easy to interpret and perform well, notably when the dataset is large. However, the problems of the model are twofold. First, the Decision Trees usually experience low prediction bias and high variance, so-called over-fitted, which means the model picks up even small perturbations and random noises in the training dataset. Furthermore, although the most optimum decision is determined at each step, this greedy model does not consider the global optimum; therefore, the overall decision tree might not be optimal. The abovementioned issues can be mitigated by ensembling methods, integrating the results of multiple trees (weak learners) into the final result (strong learner)^51,52^. Such ensemble learning algorithm in which each tree is trained in parallel forms a Decision Tree ensemble, which is referred to as Random Forests. The greedy strategy in RF determines the importance of each tree at each stage^53^. Moreover, RF can measure the feature’s importance and retain the most informative input features^54^. To improve the variable selection and diversity of the trees, the RF algorithm employs a technique called bagging or bootstrap aggregation. The model will decide how to split the input data into multiple sub-datasets according to the given trees’ population. Bagging, a type of random sampling technique, allocates a third of data for the training purpose of a subtree development process, and the remaining will be left behind, which are referred to as out-of-bag samples. Additionally, the cross-validation technique is unnecessary while using the RF algorithm as multiple bagging in the training process prevents over-fitting^55^. The framework of RF construction is illustrated in Fig. 1.

Suppose *D* is the training dataset with n number of observations, $D=[\left({x}_{1},{y}_{1}\right),\left({x}_{2},{y}_{2}\right)\cdots \left({x}_{n},{y}_{n}\right)]$, and *D*_*t*_ is the training dataset for the tree *h*_*t*_, the predicted output corresponding to the out-of-bag dataset of sample *x* can be expressed as follows^56^:

4 $${H}^{oob}\left(x\right)=argmax{\sum }_{t=1}^{T}I({h}_{t}\left(x\right))=y$$

The learning error of the OOB can be obtained by:

5 $${\varepsilon }^{oob}\left(x\right)=\frac{1}{\left|D\right|}{\sum }_{(x.y)\epsilon D}I({H}^{oob}(x)\ne y)$$

The procedure of RF must be random and this feature can be controlled over a parameter formulated as *k*^55^. The significance of a characteristic of a variable *X*_*i*_ could be obtained as follows:

6 $$I\left({X}_{i}\right)=\frac{1}{B}{\sum }_{t}^{B}{\widetilde{OOBe }}r{r}_{{t}^{i}}-OOBer{r}_{t}$$

where ${X}_{i}$ is the *i*th parameter in vector $X$, *B* indicates the current number of trees in the RF, ${\widetilde{OOB }}er{r}_{{t}^{i}}$ denotes the predicted error of the *OOB* samples for the feature ${X}_{i}$ of tree $t$, and $OOBer{r}_{t}$ is the initial *OOB* samples including permuted variables^56^.

### Decision tree (DT)

**Figure 2:**
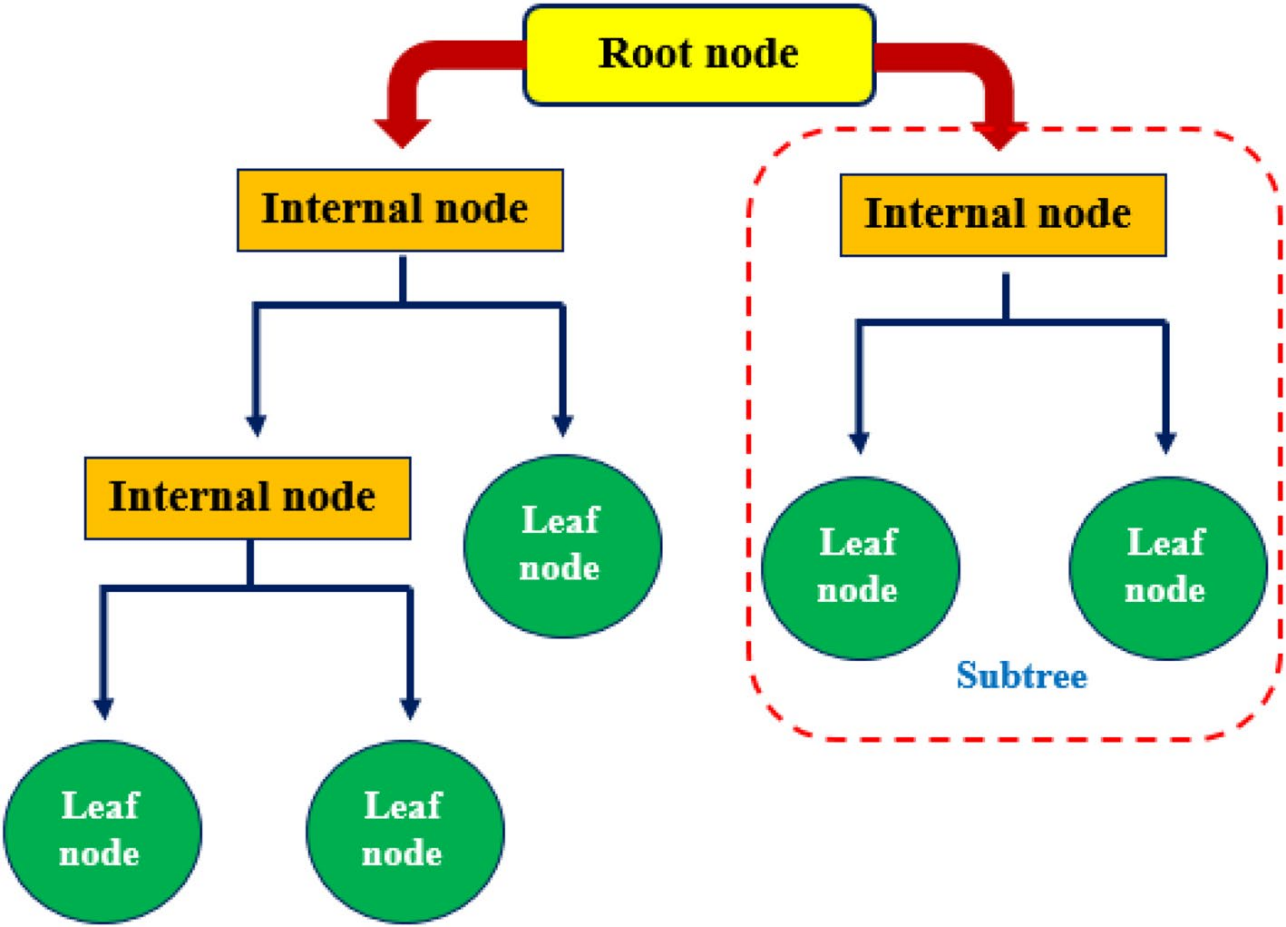
Schematic illustration of a typical decision tree.

Decision Tree, a nature-inspired supervised learning algorithm, has been widely utilized in the literature and can be used for classification and regression^57^. This algorithm consists of four elements: root node, which is the topmost node in the tree carrying the input data; leaf nodes, which are the final section of the flowchart and denotes the output of the system; internal nodes, which are placed between the root and leaf nodes; branches, which are the connection between nodes. A tree-building process in a decision tree algorithm includes three techniques: splitting, pruning, and stopping^58^. The input data is split into branches and decision nodes starting from the root node. The splitting process caries on till a stopping criterion is convinced. The pruning technique implies removing the low-importance branches^59^. A simple architecture of a DT model is illustrated in Fig. 2.

### Gradient boosting (GB)

Gradient Boosting (GB) is an effective machine learning technique that can be used in both regression and classification to reduce bias error or overfitting. Gradient boosting, as functional gradient descent, obtains the residual errors generated from the previous learner, and adds a new learner to it to minimize the loss function of the model at each stage of gradient descent. This technique aims to combine a group of weak learners in a stage-wise manner to build a strong learner and in turn, a more robust model to fit more accurately to the response variable. In other words, the new base-learner must have two conditions: be correlated with the negative gradient of the loss function and also be associated with the whole ensemble. As the idea behind gradient boosting is to minimize the loss function, there is a range of loss functions that can be used. Assume $h(x, \theta )$ is a custom base-learner and $\Psi \left(y, f\right)$ a loss function, it is tough to predict the variables and a repetitive model; therefore, is proposed to choose a new function as $h(x,{\theta }_{t})$, where the t enhancement is directed by^60,61^:

7 $${g}_{t}\left(x\right)={E}_{y}{\left[\frac{\partial \psi (y,f\left(x\right))}{\partial f(x)}\left|x\right.\right]}_{f\left(x\right)={\tilde{f }}^{t-1}(x)}$$

This converts a potential sophisticated optimization problem into a classic least square minimization^60,62^.

8 $$\left({\rho }_{t},{\theta }_{t}\right)={argmin}_{\rho ,\theta }\sum_{i=1}^{N}{\left[-{g}_{t}\left({x}_{i}\right)+\rho h({x}_{i},\theta )\right]}^{2}$$

The following are the steps in the GBDT technique process^63^:

- Suppose that ${\widehat{f}}_{0}$ is a constant
- Evaluate the ${g}_{i}(x)$ and training $h\left({x}_{i},\theta \right)$ function
- Obtain parameter ${\rho }_{i}$ and modify the function:
  9 $${\widehat{f}}_{i}={\widehat{f}}_{i-1}+{\rho }_{i}h({x}_{i},\theta )$$

**Figure 3:**
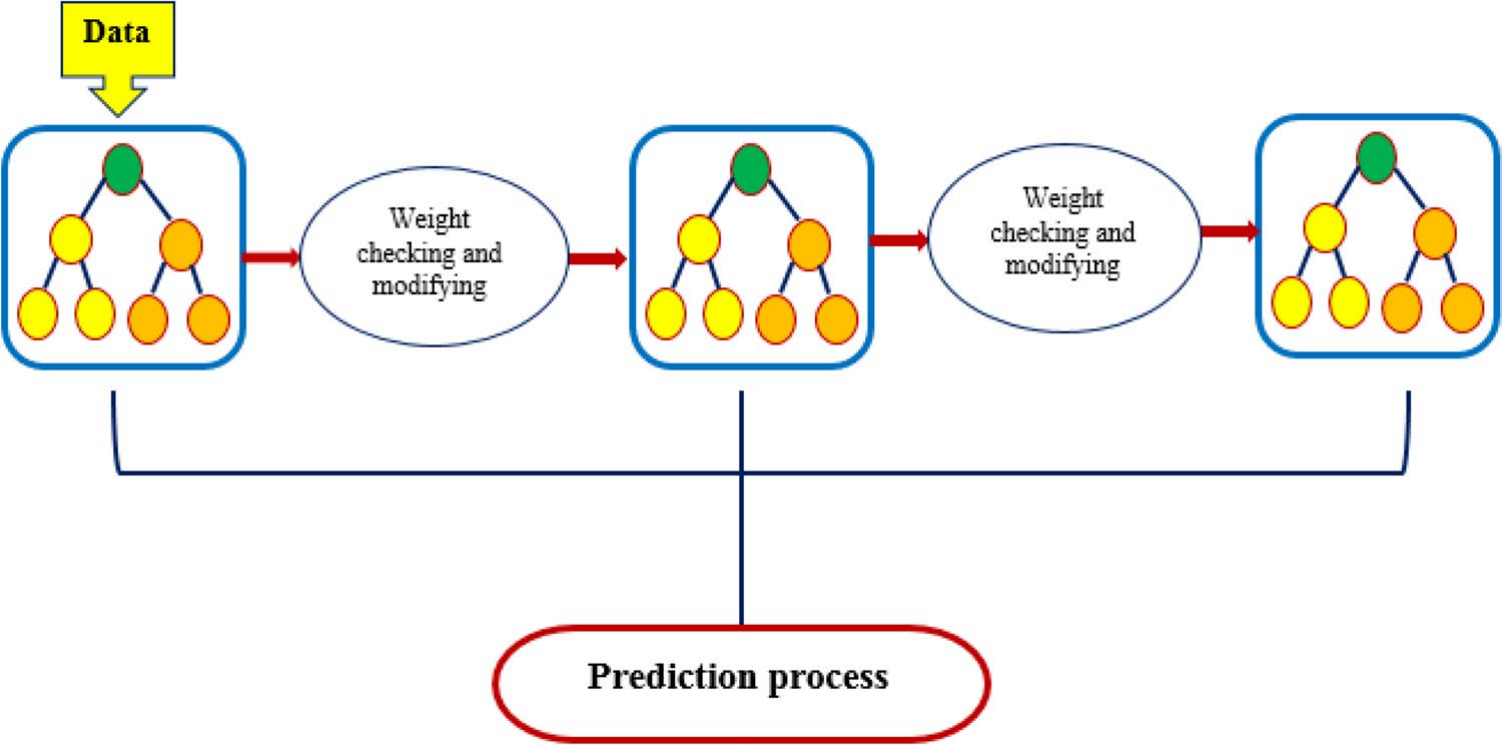
Schematic illustration of a typical GBDT.

The method starts with a single leaf and optimizes the training algorithm for each node and record. Figure 3 shows a schematic example of a conventional GBDT.

### Adaptive boosting (AdaBoost)

The adaptive boosting algorithm presented by Freund and Schapire^64^ aims to combine weak classifiers and learn from their mistake to create a strong classifier. In other words, it selects the training dataset iteratively to combine the multiple classifiers and assign the appropriate weight to each classifier based on the accuracy of each classifier so that higher weights are assigned to the misclassified/mislabeled samples^65^. The following are the general stages of the AdaBoost technique^66,67^:

- Weights definition: $${w}_{j}=\frac{1}{n},\, j=1,2,\dots ,n$$
- Apply the training data to a weak learner *Wl*_*i*_* (x)*, weights, and obtain the weighted error for each *i*.
  10 $$I\left(x\right)=\left\{\begin{array}{ll}0 &\quad if\, x=false\\ 1 &\quad if\, x=true\end{array}\right.$$
  11 $${Err}_{i}=\frac{{\sum }_{j=1}^{n}{w}_{j}I({t}_{j}\ne {wl}_{i}\left(x\right))}{{\sum }_{j=1}^{n}{w}_{j}}$$
- Determine the weights for predictors for each i as follows:
  12 $${\beta }_{i}=log\left(\frac{(1-{Err}_{i})}{{Err}_{i}}\right)$$
- Update the sample weights for each *i* to *N* (where *N* is the learner's number)
- Assign a weak learner to the data test (*x*) as a result.

Support vector regressors (SVR) and Decision Trees (DT) have been used as weak learners in AdaBoost systems in this study.

In this paper, we have applied ensemble models such as Adaboost-DT, Adaboost-DT, and GB-SVR. To discover the functionality and different possibilities of regression methods, AdaBoost and Gradient boosting as varieties of clustering methods have been executed to enhance the conventional weak regressors by incorporating the outcome of the weak regressors into a weighted combination that determines the best output of the enhanced powerful regressor and also the outcome of the weak regressors is distorted in pursuit of incorrectly estimated samples autonomously.

More details are as follows:

Linear SVR indistinguishability is achieved by using a nonlinear imaging approach to convert features with linearly unidentifiable low-dimensional input space into a high-dimensional feature space. This allows the nonlinear features of the samples to be analyzed linearly using a linear algorithm in a high-dimensional feature space. However, the choice of kernel functions and parameters has a significant impact on its performance^68^. The AdaBoost method trains many base learners, and the sample generalization could be further improved by combining techniques to produce the final strong learner. Anomaly samples are susceptible to the AdaBoost method, and anomalous samples may obtain greater weights in iterations, affecting the prediction accuracy of strong learners. Furthermore, the decision tree is widely used as a basic learning method, but it is inadequate in dealing with nonlinear issues, and prediction accuracy varies substantially^69^. The AdaBoost method, on the other hand, is sensitive to anomalous data, and anomalous samples may obtain greater weights in iterations, affecting strong learners' prediction accuracy.

When using SVR for sample learning, the model's performance is determined by the kernel function and kernel parameters. Using SVR as the AdaBoost base learner, on the other hand, lowers the influence of the SVR algorithm's kernel functions and parameters. It also overcomes AdaBoost's standard algorithm's inability to address nonlinear issues. This makes the AdaBoost-SVR method appropriate for dealing with nonlinear feature data prediction while also ensuring the model's generalizability^70^. We combined GB and SVR algorithms^71^. The combined GB and SVR algorithm into a single predictive model is another meta-algorithm applied in this paper in order to enhance the overall performance. Gradient Boosting as part of an ensemble technique attempts to create a strong regressor from several weak regressors.

### Equations of state (EOS)

An EOS is a mathematical representation that connects system parameters to represent the state of a material under a range of predefined circumstances, including pressure, temperature, or volume^72^. These thermodynamic models can characterize the thermal characteristics and volumetric behavior of mixtures and pure materials^73^. During the last few decades, cubic EOSs have been widely employed. New EOSs, like various forms of the Statistical Associating Fluid Theory (SAFT), have been applied successfully in the past few years. To explain the interactions between the molecules in a system, the SAFT EOSs were constructed using statistical mechanics^74,75^. SAFT EOSs are designed to depict molecules as chains of spherical particles that engage with others via long-range attraction, short-range repulsion, and hydrogen bonding at particular places. In this study, four equations of state such as PR, SRK, VPT, and PC-SAFT, have been used. The PVT relationships and parameters of the respective equations of state are reported in Tables 2 and 3. The critical properties and acentric coefficients of the materials utilized in this study are summarized in Table 4.

**Table 2:** EOSs relationships and parameters.

EOS	PVT relation	Parameters	Reference
SRK	$P = \frac{RT}{{\nu - b}} - \frac{{a_{c} \alpha }}{\nu (\nu + b)}$	$$\begin{gathered} a_{C} = {0}{\text{.42747}}\frac{{R^{2} T_{C}^{2} }}{{P_{C} }} \hfill \\ b = {0}{\text{.08664}}\frac{{RT_{C} }}{{P_{C} }} \hfill \\ m = 0.480 + 1.574\omega - 0.176\omega^{2} \hfill \\ \alpha = \left[ {1 + m\left( {1 - \sqrt {T_{r} } } \right)} \right]^{2} \hfill \\ \end{gathered}$$	^76^
PR	$P = \frac{RT}{{\nu - b}} - \frac{\alpha }{{\nu (\nu + b) + b\left( {\nu - b} \right)}}$	$$\begin{gathered} \alpha = a_{C} \alpha (T) \hfill \\ \alpha (T) = \left[ {1 + m\left( {1 - \sqrt {T_{r} } } \right)} \right]^{2} \hfill \\ a_{C} = 0.45724\frac{{(RT_{C} )^{2} }}{{P_{C} }} \hfill \\ m = 0.37464 + 1.54226\omega - 0.26992\omega^{2} \hfill \\ b = {0}{\text{.07780}}\frac{{RT_{C} }}{{P_{C} }} \hfill \\ \end{gathered}$$	^76^
VPT	$P = \frac{RT}{{\nu - b}} - \frac{a(T)}{{\nu (\nu + b) + c\left( {\nu - b} \right)}}$	$$\begin{aligned} & a(T) = {\text{a}}^{{{\text{C }}}} {\text{ + a}}^{{\text{A}}} \\ & {\text{a}}^{{{\text{C }}}} = \sum\limits_{i} {\sum\limits_{j} {x_{i} x_{j} \sqrt {a_{i} a_{j} } } } (1 - k_{{i_{j} }} ) \\ & {\text{a}}^{{\text{A}}} = \sum\limits_{p} {x_{p}^{2} } \sum\limits_{i} {x_{i} a_{{pi}} l_{{pi}} } \\ & a_{{pi}} = \sqrt {a_{p} a_{i} } \\ & l_{{pi}} = l_{{pi}}^{0} - l_{{pi}}^{1} (T - 273.15) \\ \end{aligned}$$	^77–79^
PC-SAFT	$\tilde{a }=\frac{A}{kTN}=$ ${\tilde{a }}^{hc}+{\tilde{a }}^{id}+{\tilde{a }}^{disp}+{\tilde{a }}^{assoc}$	$$\begin{gathered} {\tilde{\text{a}}}^{{{\text{hc}}}} = \overline{m}{\tilde{\text{a}}}^{{{\text{hs}}}} + {\tilde{\text{a}}}^{{{\text{chain}}}} = \overline{m}{\tilde{\text{a}}}^{{{\text{hs}}}} - \sum\limits_{i} {x_{i} } (m_{i} - 1)\ln g_{ij}^{hs} \hfill \\ \overline{m} = \sum\limits_{i} {x_{i} } m_{i} \hfill \\ {\tilde{\text{a}}}^{{{\text{hs}}}} = \frac{1}{{\zeta_{0} }}\left[ {\frac{{3\zeta_{1} \zeta_{2} }}{{1 - \zeta_{3} }} + \frac{{3\zeta_{2}^{3} }}{{\zeta_{3} (1 - \zeta_{3} )^{2} }} + \left( {\frac{{\zeta_{2}^{3} }}{{\zeta_{3}^{2} }} - \zeta_{0} } \right)\ln (1 - \zeta_{3} )} \right] \hfill \\ \zeta_{n} = \frac{\pi }{6}\rho \sum\limits_{i} {x_{i} } m_{i} d_{i}^{n} \, n \in \left\{ {0,1,2,3} \right\}, \, \eta = \zeta_{3} \hfill \\ d_{i} = \sigma_{i} \left[ {1 - 0.12\exp \left( { - 3\frac{{\varepsilon_{i} }}{kT}} \right)} \right] \hfill \\ g_{ij}^{hs} = \frac{1}{{1 - \zeta_{3} }} + \left( {\frac{{d_{i} d_{j} }}{{d_{i} + d_{j} }}} \right)\frac{{2\zeta_{2} }}{{(1 - \zeta_{3} )^{2} }} + \left( {\frac{{d_{i} d_{j} }}{{d_{i} + d_{j} }}} \right)^{2} \frac{{2\zeta_{2}^{2} }}{{(1 - \zeta_{3} )^{2} }} \hfill \\ {\tilde{\text{a}}}^{{{\text{dis}}}} = - 2\pi \rho I_{1} \left( {\eta ,\overline{m}} \right)\overline{{m^{2} \varepsilon \sigma^{3} }} - \pi \rho \overline{m}C_{1} \left( {\eta ,\overline{m}} \right)I_{2} \left( {\eta ,\overline{m}} \right)\overline{{m^{2} \varepsilon^{2} \sigma^{3} }} \hfill \\ I_{1} \left( {\eta ,\overline{m}} \right) = \sum\limits_{i = 0}^{6} {a_{i} } (\overline{m})\eta^{i} { , }I_{2} \left( {\eta ,\overline{m}} \right) = \sum\limits_{i = 0}^{6} {b_{i} } (\overline{m})\eta^{i} \hfill \\ \end{gathered}$$ where *a*_*i*_ and *b*_*i*_ depend on the chain length as given in Gross and Sadowski^80^ $$\begin{gathered} C_{1} = \left[ {1 + \overline{m}\frac{{8\eta - 2\eta^{2} }}{{\left( {1 - \eta } \right)^{4} }} + (1 - \overline{m})\frac{{20\eta - 27\eta^{2} + 12\eta^{3} - 2\eta^{4} }}{{\left[ {\left( {1 - \eta } \right)\left( {2 - \eta } \right)} \right]^{2} }}} \right] \hfill \\ \overline{{m^{2} \varepsilon \sigma^{3} }} = \sum\limits_{i} {\sum\limits_{j} {x_{i} x_{j} } } m_{i} m_{j} \left( {\frac{{\varepsilon_{ij} }}{kT}} \right)\sigma_{ij}^{3} \hfill \\ \overline{{m^{2} \varepsilon^{2} \sigma^{3} }} = \sum\limits_{i} {\sum\limits_{j} {x_{i} x_{j} } } m_{i} m_{j} \left( {\frac{{\varepsilon_{ij} }}{kT}} \right)^{2} \sigma_{ij}^{3} \hfill \\ \varepsilon_{ij} = \sqrt {\varepsilon_{i} \varepsilon_{j} } \left( {1 - k_{ij} } \right) \hfill \\ \sigma_{ij} = \frac{{\left( {\sigma_{i} + \sigma_{j} } \right)}}{2} \hfill \\ \end{gathered}$$ The formulation for the contributions from the dispersion and ideal gas are similar to those of Gross and Sadowski^80^	^80,81^

**Table 3:** PC-SAFT EOS factors for the substances utilized in this paper.

Substance	*M*_*w*_ (g/mol)	*m*	*σ* (Å)	*ε/k* (K)	Reference
N_2_	28.013	1.2053	3.313	90.96	^80^
CO_2_	44.01	2.0729	2.7852	169.21	^80^
H_2_O	18.015	2	2.3533	207.84	^82^

**Table 4:** Critical properties and acentric factors utilized in the EOSs for the substances used in this paper^79^.

Substance	P_c_ (MPa)	T_c_ (K)	Z_c_	ω
N_2_	3.394	126.10	0.2917	0.0403
CO_2_	7.382	304.19	0.2744	0.2276
H_2_O	22.055	647.13	0.2294	0.3449

## Performance analysis of models

The mathematical description of the statistical parameters employed in this study are summarized below^72,83^:

- Average absolute percent relative error (AAPRE)
  13 $$AAPRE= \frac{1}{N}\sum_{i=1}^{N}\left|\left(\left({S}_{i EXP}-{S}_{i PRED}\right)/{S}_{i EXP}\right)\times 100\right|$$
- Standard deviation (SD)
  14 $$SD=\sqrt{\frac{1}{N-1}\sum_{i=1}^{N}{\left(\frac{{S}_{i EXP}-{S}_{i PRED}}{{S}_{i EXP}}\right)}^{2}}$$
- Coefficient of determination (R^2^)
  15 $${R}^{2}=1-\frac{\sum_{i=1}^{N}{\left({S}_{i EXP}-{S}_{i PRED}\right)}^{2}}{\sum_{i=1}^{N}{\left({S}_{i EXP}-\overline{{S }_{i EXP}}\right)}^{2}}$$
- Root mean square error (RMSE)
  16 $$RMSE= \sqrt{\frac{1}{N}\sum_{i=1}^{N}{\left({S}_{i EXP}-{S}_{i PRED}\right)}^{2}}$$

In the above equations, ${S}_{i EXP}$, ${S}_{i PRED}$, $\overline{{S }_{i EXP}}$, and *N* refer to experimental solubility, predicted solubility, mean experimental solubility, and the total number of data points, respectively.

Also, several graphical analyses, namely, cross-plot, relative error distribution diagram, cumulative frequency plot, and trend plot were utilized to visually evaluate the developed models. Descriptions of these analyses can be found elsewhere^72^.

## Results and discussion

### Statistical evaluation of models

The models discussed in the previous sections have been developed to predict the solubility of CO_2_–N_2_ mixtures in water utilizing 289 laboratory data. In this study, we have employed six algorithms, which were rarely used, to estimate CO_2_–N2 gas mixture solubility in water. The structure of the models was modified and also the grid search algorithm was used to optimize the hyperparameters of the models to avoid overfitting in this particular problem. The hyperparameters obtained by the grid search are different for each model. It is based on the importance of the hyperparameters according to theoretical and practical aspects. Total data has been divided randomly to 80/20 for the training and testing phase. It should be noted that experimental data and predictions of different models are presented in the Supplementary file. The calculated statistical parameters for the represented models are summarized in Table 5. In this table, different statistical parameters such as RMSE, R^2^, SD, and AAPRE are reported. GB-SVR outperforms other models except for Random Forest because SVR is more like a soft fabric that can bend and fold in whatever way we need to better fit our data. This gives more degrees of freedom and flexibility so that a more accurate model can be achieved. Moreover, SVR can capture the non-linear relationships between variables. The performance of the model is further improved by tuning the hyperparameters. These are the main reasons that GB-SVR has shown a higher accuracy. Random Forest proved the highest accuracy in this study even higher than SVR-GB. Random Forest is built for multiclass issues, whereas SVM is for two-class problems. In SVM, in the case of a multiclass problem, the problem must be broken down into numerous binary classification tasks. With a combination of numerical and categorical variables, Random Forest performs well. Also, in classification problems, it is not necessary to do normalization or scaling in Random Forest. SVM seeks to maximize the "margin," relying on the idea of "distance" between points. It is up to us to decide if "distance" is significant. As a consequence, one-hot encoding for categorical features is a must-do. Further, min–max or other scaling is highly recommended in preprocessing step. Random forests are good for a specific set of issue types when given a specific set of data, but they do not act well for many others. We should mention that random forests are unexpectedly effective for a wide range of issues because they are built on trees, the variables cannot be scaled. A tree inherently captures any monotonic alteration of a single variable, and in random forest built-in feature selection is automated^84^.

**Table 5:** Calculated statistical criteria for the proposed models.

Statistical criteria		RMSE	SD	R^2^	AAPRE (%)
DT	Train	0.000297	0.1044	0.9978	6.1904
	Test	0.000290	0.3172	0.9965	12.3069
	Total	0.000295	0.1721	0.9977	7.4179
GB-DT	Train	0.000166	0.2324	0.9993	10.5323
	Test	0.000155	0.3973	0.9991	15.3978
	Total	0.000164	0.2745	0.9992	11.5088
AdaBoost-DT	Train	0.000217	0.2331	0.9988	12.5086
	Test	0.000204	0.2901	0.9985	13.7655
	Total	0.000214	0.2457	0.9987	12.7609
AdaBoost-SVR	Train	0.000161	0.2220	0.9993	9.5464
	Test	0.000147	0.2076	0.9992	9.6933
	Total	0.000159	0.2192	0.9993	9.5759
GB-SVR	Train	0.000300	0.1120	0.9977	6.7068
	Test	0.000290	0.0716	0.9970	5.3403
	Total	0.000298	0.1051	0.9976	6.4326
Random forest	Train	0.000132	0.0740	0.9995	2.9086
	Test	0.000131	0.0608	0.9994	2.5999
	Total	0.000132	0.0716	0.9995	2.8466

According to Table 5, it can be seen that the Random Forest model with an AAPRE value of 2.84% has the most accurate prediction for the solubility of CO_2_–N_2_ mixtures in water. The GB-SVR and DT models with AAPRE values of 6.43% and 7.41%, respectively, have the closest prediction to the Random Forest model compared to other models. However, it should be noted that other models also have relatively good results. Another noteworthy point is that sometimes the high accuracy of a model in predicting outputs may be due to over-training. In order to ensure that this does not happen, the results of training and test data should be compared with each other. If the difference between the statistical parameters of the training and test data is significant, the model may be over-trained. If the results of the training and test data are close to each other, it can be stated that over-training has not happened. As the results show, the statistical parameters for the training and test data are very close.

To evaluate the performance of artificial intelligence methods in comparison with mathematical methods, four equations of state such as SRK, PR, VPT, and PC-SAFT, have been used. For this purpose, the solubility of CO_2_ and N_2_ in different CO_2_ + N_2_ + H_2_O (brine) systems was calculated using 24 laboratory data points extracted from the literature^10^, and the results are reported in Table 6 and Table 7. As shown in Tables 6 and 7, the value of AAPRE obtained for the SRK and PR equations of state is much higher than VPT and PC-SAFT equations of state and the intelligent models. For solubility of CO_2_ in aqueous solutions, the Random Forest approach outperforms the other intelligent techniques with an AAPRE value of 1.16%, and the PC-SAFT model has the best results among the EOSs with an AAPRE value of 3.35%. For solubility of N_2_ in aqueous solutions, the Random Forest technique has the best results among the intelligent approaches with an AAPRE value of 4.13%, and the VPT model has the best results among the EOSs with an AAPRE value of 5.71%.

**Table 6:** Predictions of EOSs and smart models for CO_2_ solubility in different CO_2_ + N_2_ + H_2_O (brine) systems.

Solubility system	Data no.	P (MPa)	CO_2_ solubility (mole fraction)										
			Exp	DT	GB-DT	AdaBoost-DT	AdaBoost-SVR	GB-SVR	Random Forest	SRK	PR	VPT	PC-SAFT
CO_2_ (14.6%) + N_2_ (85.4%) + H_2_O, at 303.05 K	1	1.98	0.0008	0.000791	0.000800	0.000900	0.000900	0.000816	0.000800	0.0010	0.0013	0.0009	0.0008
	2	5.63	0.0022	0.002101	0.002200	0.002150	0.002200	0.002095	0.002200	0.0023	0.0032	0.0023	0.0021
	3	9.35	0.0031	0.003384	0.003350	0.003542	0.003460	0.003258	0.003050	0.0033	0.0041	0.0033	0.0031
	4	13.17	0.0039	0.003888	0.003900	0.003975	0.003900	0.003794	0.003750	0.0039	0.0049	0.0043	0.0038
	5	16.97	0.0045	0.004417	0.004500	0.004500	0.004500	0.004444	0.004650	0.0045	0.0061	0.0048	0.0043
	6	20.75	0.0048	0.004717	0.004575	0.004560	0.004575	0.004718	0.004650	0.0051	0.0066	0.0053	0.0046
CO_2_ (3%) + N_2_ (97%) + H_2_O, at 283.15 K	7	2.05	0.0003	0.000405	0.000400	0.000780	0.000400	0.000410	0.000300	0.0002	0.0003	0.0003	0.0003
	8	5.74	0.0008	0.001142	0.000800	0.000900	0.000800	0.001135	0.000800	0.0005	0.0007	0.0008	0.0008
	9	9.84	0.0012	0.001258	0.001400	0.001400	0.001400	0.001241	0.001200	0.0007	0.0010	0.0013	0.0012
	10	13.58	0.0014	0.001501	0.001600	0.001700	0.001500	0.001477	0.001400	0.0008	0.0012	0.0015	0.0014
	11	18.06	0.0017	0.001739	0.001700	0.001750	0.001800	0.001739	0.001700	0.0010	0.0014	0.0018	0.0016
	12	21.5	0.0018	0.001843	0.001800	0.001800	0.001800	0.001828	0.001800	0.0011	0.0016	0.0021	0.0017
CO_2_ (61%) + _2_N (39%) + H_2_O, at 303.05 K	13	1.92	0.0024	0.003120	0.002400	0.002400	0.002400	0.003257	0.002400	0.0032	0.0057	0.0026	0.0024
	14	5.59	0.0064	0.007550	0.006400	0.006400	0.006400	0.008086	0.006400	0.0093	0.0099	0.0065	0.0062
	15	9.06	0.0091	0.009971	0.009100	0.009100	0.009100	0.009878	0.009100	0.0113	0.0124	0.0091	0.0087
	16	13.3	0.0112	0.011830	0.011200	0.011400	0.011467	0.011521	0.011200	0.0133	0.0144	0.0113	0.0107
	17	17.03	0.012	0.012651	0.012000	0.012000	0.012000	0.012598	0.012250	0.0142	0.0178	0.0125	0.0117
	18	20.99	0.0125	0.013007	0.012500	0.012500	0.012500	0.012988	0.012250	0.0156	0.0201	0.0133	0.0123
CO_2_ (14.6%) + N_2_ (85.4%) + brine (10 wt.% NaCl), at 273.25 K	19	2	0.0013	0.001176	0.001300	0.001200	0.001250	0.001186	0.001300	0.0011	0.0016	0.0013	0.0013
	20	5.59	0.0033	0.003190	0.003225	0.003175	0.003300	0.003184	0.003300	0.0025	0.0036	0.0032	0.0031
	21	9.42	0.0047	0.004564	0.004665	0.004462	0.004733	0.004561	0.004700	0.0034	0.0048	0.0046	0.0044
	22	13.26	0.0056	0.005460	0.005600	0.005428	0.005600	0.005432	0.005900	0.0041	0.0055	0.0055	0.0052
	23	17.12	0.0061	0.005996	0.006000	0.005800	0.006002	0.005914	0.006500	0.0053	0.0058	0.0063	0.0056
	24	21.08	0.0065	0.006276	0.006079	0.005900	0.006129	0.006212	0.006500	0.0058	0.0061	0.0066	0.0059
AAPRE, %		–	–	8.71	3.67	11.87	4.42	9.03	1.16	23.34	30.18	5.08	3.35

**Table 7:** Predictions of EOSs and smart models for N_2_ solubility in different CO_2_ + N_2_ + H_2_O (brine) systems.

Solubility system	Data no.	P (MPa)	N_2_ solubility (mole fraction)										
			Exp	DT	GB-DT	AdaBoost-DT	AdaBoost-SVR	GB-SVR	Random Forest	SRK	PR	VPT	PC-SAFT
CO_2_ (14.6%) + N_2_ (85.4%) + H_2_O, at 303.05 K	1	1.98	0.0002	0.000196	0.000300	0.000300	0.000300	0.000195	0.000200	0.0002	0.0003	0.0002	0.0002
	2	5.63	0.0005	0.000498	0.000611	0.000600	0.000500	0.000486	0.000660	0.0005	0.0007	0.0005	0.0005
	3	9.35	0.0008	0.000802	0.000900	0.000900	0.000900	0.000819	0.000750	0.0007	0.0011	0.0008	0.0007
	4	13.17	0.0011	0.001034	0.001200	0.001100	0.001100	0.001044	0.001000	0.0010	0.0013	0.0011	0.0009
	5	16.97	0.0013	0.001269	0.001350	0.001367	0.001300	0.001223	0.001300	0.0013	0.0016	0.0014	0.0011
	6	20.75	0.0015	0.001471	0.001500	0.001500	0.001500	0.001485	0.001500	0.0015	0.0019	0.0016	0.0013
CO_2_ (3%) + N_2_ (97%) + H_2_O, at 283.15 K	7	2.05	0.0003	0.000331	0.000400	0.000320	0.000300	0.000338	0.000300	0.0002	0.0004	0.0003	0.0003
	8	5.74	0.0008	0.000790	0.000800	0.000750	0.000800	0.000793	0.000748	0.0004	0.0009	0.0008	0.0007
	9	9.84	0.0012	0.001235	0.001213	0.001200	0.001200	0.001247	0.001200	0.0007	0.0012	0.0012	0.0011
	10	13.58	0.0016	0.001632	0.001600	0.001600	0.001600	0.001625	0.001600	0.0009	0.0018	0.0016	0.0014
	11	18.06	0.002	0.002035	0.002000	0.002000	0.002000	0.002050	0.002000	0.0011	0.0024	0.002	0.0018
	12	21.5	0.0023	0.002332	0.002300	0.002100	0.002300	0.002329	0.002300	0.0013	0.0027	0.0023	0.0020
CO_2_ (61%) + N_2_ (39%) + H_2_O, at 303.05 K	13	1.92	0.0001	0.000157	0.000307	0.000200	0.000200	0.000163	0.000100	0.0001	0.0002	0.0002	0.0001
	14	5.59	0.0004	0.000430	0.000600	0.000600	0.000440	0.000433	0.000354	0.0003	0.0005	0.0004	0.0004
	15	9.06	0.0006	0.000613	0.000600	0.000600	0.000600	0.000640	0.000560	0.0004	0.0008	0.0006	0.0006
	16	13.3	0.0008	0.000816	0.000800	0.000800	0.000800	0.000842	0.000727	0.0006	0.0011	0.0009	0.0008
	17	17.03	0.001	0.001079	0.001000	0.001000	0.001000	0.001019	0.001000	0.0008	0.0013	0.0011	0.0009
	18	20.99	0.0012	0.001268	0.001200	0.001200	0.001200	0.001265	0.001200	0.0010	0.0017	0.0012	0.0011
CO_2_ (14.6%) + N_2_ (85.4%) + brine (10 wt.% NaCl), at 273.25 K	19	2	0.0002	0.000172	0.000200	0.000200	0.000200	0.000171	0.000200	0.0001	0.0003	0.0002	0.0001
	20	5.59	0.0004	0.000420	0.000500	0.000484	0.000400	0.000424	0.000328	0.0003	0.0007	0.0004	0.0004
	21	9.42	0.0006	0.000615	0.000650	0.000800	0.000600	0.000608	0.000600	0.0005	0.0009	0.0006	0.0006
	22	13.26	0.0008	0.000776	0.000800	0.000850	0.000800	0.000776	0.000800	0.0007	0.0011	0.0008	0.0008
	23	17.12	0.001	0.000977	0.001000	0.001100	0.001000	0.000979	0.001000	0.0009	0.0014	0.001	0.0009
	24	21.08	0.0012	0.001134	0.001200	0.001200	0.001200	0.001158	0.001200	0.0011	0.0016	0.0012	0.0010
AAPRE, %		–	–	6.13	17.59	13.74	7.18	6.81	4.13	21.72	35.18	5.71	8.78

### Graphical analysis of models

**Figure 4:**
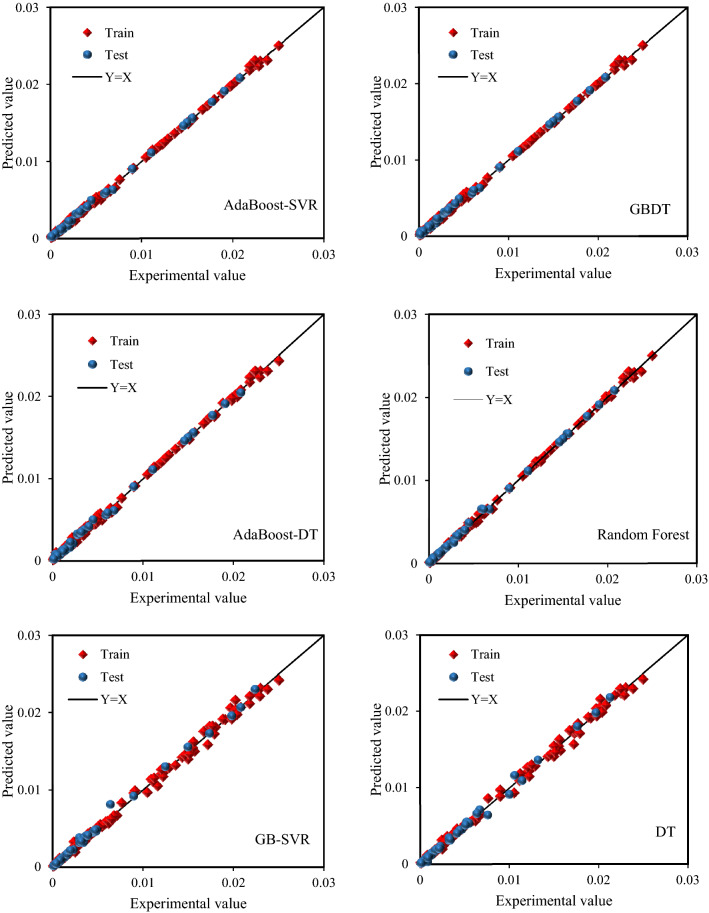
Cross plots of the developed models in this study.

Figure 4 shows the cross-plot diagrams for the six models presented in this study. In this graph, where the predicted results are plotted against actual values, the higher the compaction of data around the Y = X line indicates that the estimated values are closer to the actual values; therefore, the model is more accurate. In addition, R^2^ value for this dataset will close to 1. As shown in Fig. 4, the Random Forest model is in a better position than the other models, which also confirms the results reported in Table 5.

**Figure 5:**
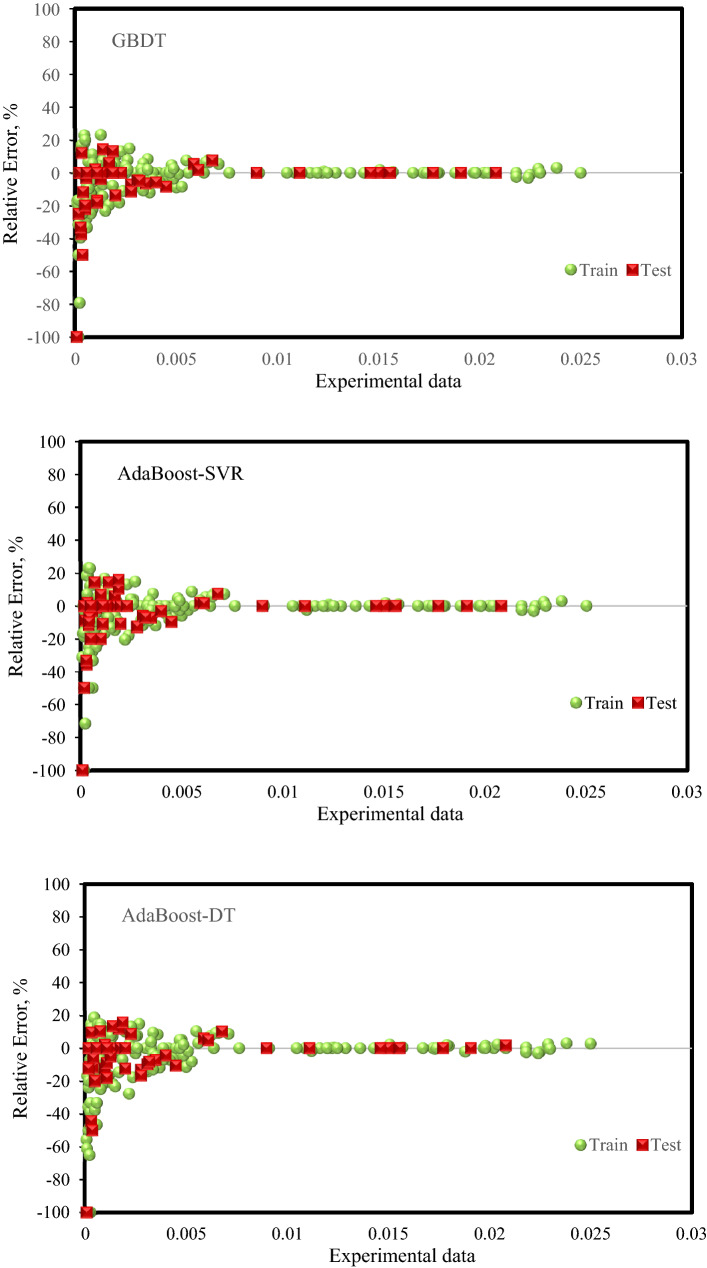

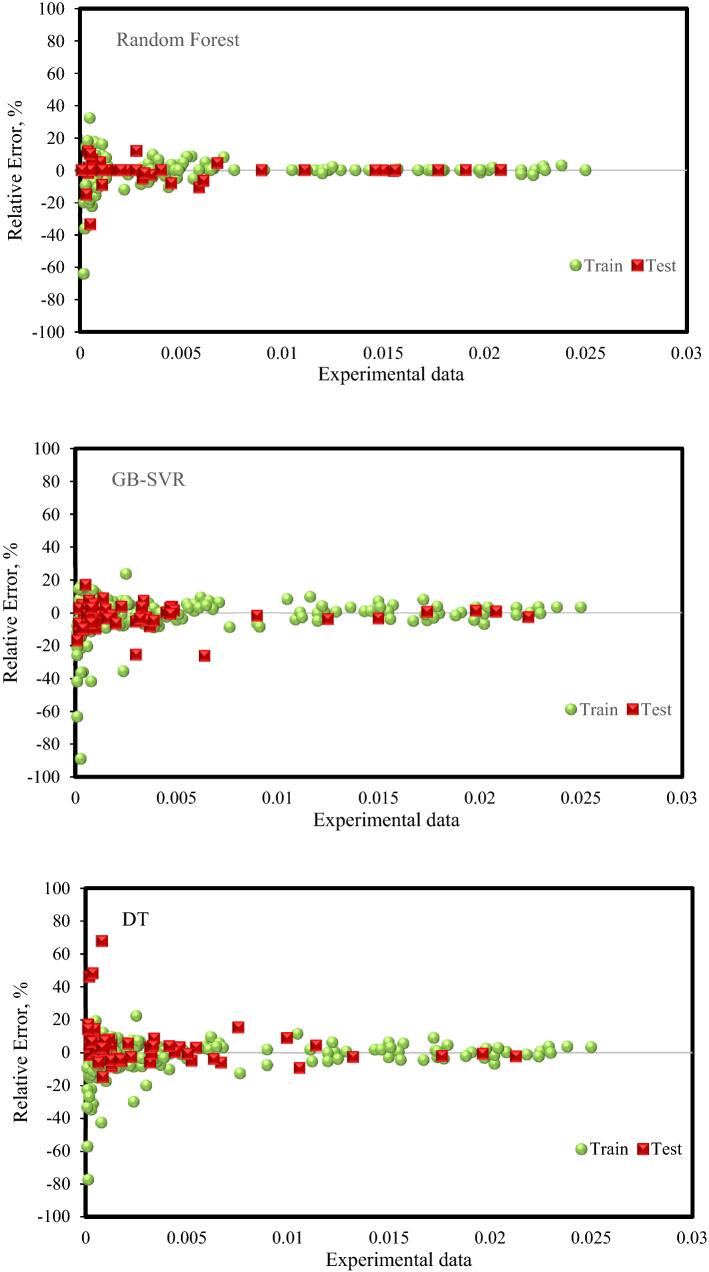
Error distribution plots of the developed models for training and test sets.

Figure 5 shows the error distribution diagram for the developed models. This diagram shows the relative error on the Y-axis and the experimental data on the X-axis. The closer and the more compaction of the points around the zero line, the less the predicted data error. On the other hand, according to this diagram, the relative error range for experimental data can be visually observed. For example, it can be seen how the relative error will change as the value of experimental data increases. As shown in Fig. 5, it can be observed that the Random Forest model is in a better condition and shows relatively lower errors than other models.

**Figure 6:**
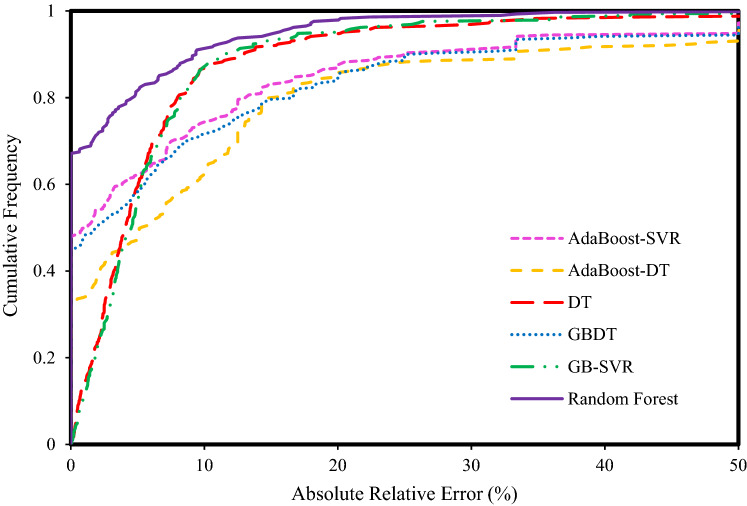
The cumulative frequency plot for the developed predictive models.

A cumulative frequency graph is one of the most important diagrams that can be used to compare the performance of several models simultaneously. Figure 6 shows a cumulative frequency diagram for different models. In this diagram, which is a cumulative frequency of the number of data in terms of absolute relative error, the higher the curve of one model than the curve of other models, the higher the accuracy. In other words, if a model's curve is higher than another model's curve in a constant AAPRE value, it means that a higher percentage of the data in that model has a lower absolute relative error than another model. The higher the curve of one model at small absolute relative errors (close to 1), the higher the percentage of that data, the lower the absolute relative error, and the more accurate the model. Therefore, according to Fig. 6 and what is said, the Random Forest model is in a better situation than other models and has a higher accuracy, which also confirms the results presented in Table 5.

### Trend analysis

Investigating the trend of solubility changes in terms of different parameters can give us a better understanding of the solubility of CO_2_–N_2_ mixture in water and brine solutions. On the other hand, the validity of the developed models can be investigated by comparing the trend of measured changes with laboratory data, equations of state, and predicted data. For example, when an input parameter shows an increasing trend in experimental data, the developed models should show the same trend. In this case, the validity of the developed model will be more. In the following, we examined the trend analysis of various parameters.

**Figure 7:**
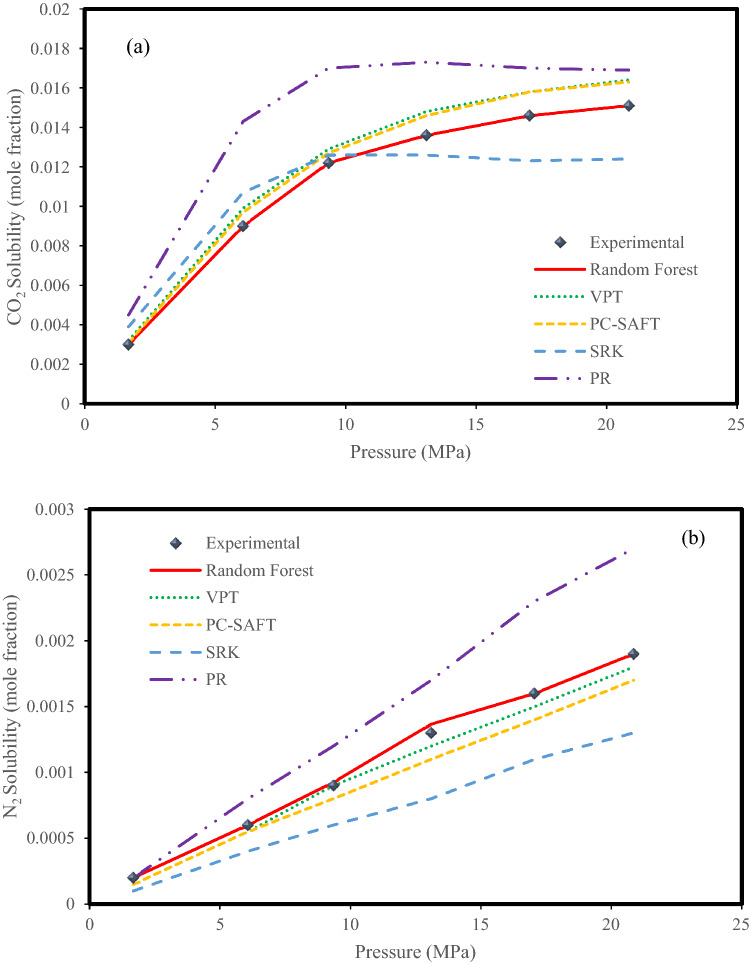
Experimental values^10^ with predictions of the EOSs and Random Forest model for the (**a**) CO_2_ solubility and (**b**) N_2_ solubility in the N_2_ (39%) + CO_2_ (61%) + H_2_O system at a temperature of 283 K.

Figure 7 shows the effect of pressure on the solubility of CO_2_ and N_2_ in an aqueous system consisting of 39% N_2_ and 61% CO_2_ at 283 K. In this figure, the changes in solubility in terms of pressure using laboratory and predicted data in the Random Forest model as the best model and equations of state were investigated. According to Fig. 7a and b, all methods show an incremental trend. What is debatable in this figure is the degree to which the models are overestimated and underestimated. Another noteworthy point is the perfect agreement of the Random Forest model data with the experimental data, which confirms the efficiency of the intelligent models. As shown in Fig. 7a, the curves related to the equations of state are generally in a higher position than the curve of the experimental data, and this indicates that these equations overestimate the solubility of CO_2_ in the mentioned system. Figure 7b also shows the conformity of the data curve predicted by the Random Forest model with the experimental data, but the different point is that the PR EOS overestimates the solubility of N_2_ in the mentioned system and other models underestimate although the degree of agreement of the VPT EOS to the experimental data is significant. Again, for solubility of CO_2_ present in gaseous mixtures in aqueous systems, the PC-SAFT model, and for solubility of N_2_, the VPT model had the best results among the EOSs.

**Figure 8:**
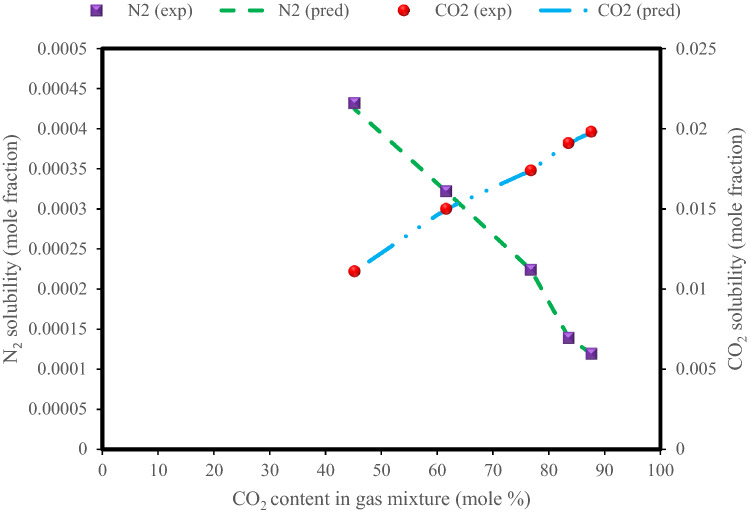
Dependence of CO_2_ and N_2_ solubilities on the mole percent of CO_2_ in the gaseous phase in the N_2_ + CO_2_ + H_2_O system at a temperature of 308 K and pressure of 8.0 MPa.

Figure 8 shows the effect of CO_2_ content in the gas mixture for the solubility of CO_2_ and N_2_ in an aqueous system containing CO_2_ and N_2_ at a temperature of 308 K and pressure of 8 MPa, as experimentally investigated in the literature^18^. As expected, increasing the amount of CO_2_ in the gas mixture reduces the solubility of N_2_ in the system and, conversely, increases the solubility of CO_2_ at constant temperature and pressure. As it is clear, the solubility of N_2_ in water is less than that of CO_2_.

**Figure 9:**
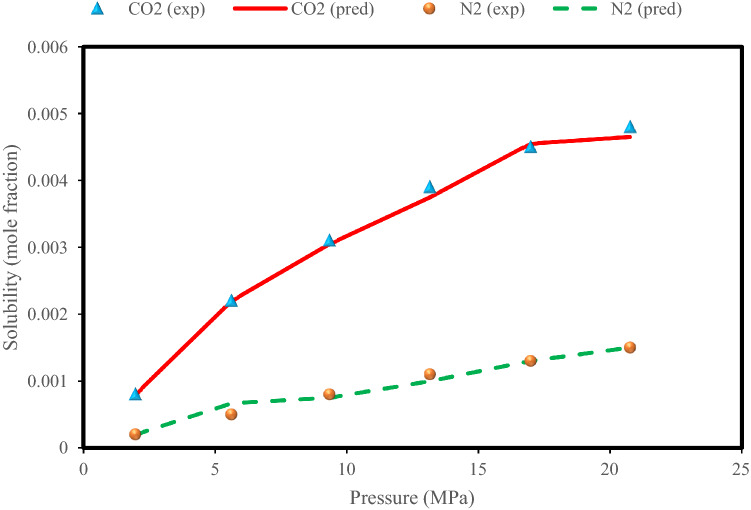
Experimental values^10^ of CO_2_ and N_2_ solubilities in the N_2_ (85.4%) + CO_2_ (14.6%) + H_2_O system at a temperature of 303 K with predictions of the Random Forest model.

Figure 9 shows the effect of pressure on the solubility of CO_2_ and N_2_ in a system containing 85.4% N_2_ and 14.6% CO_2_ in water at 303 K for the Random Forest model and laboratory data^10^. As shown in Fig. 9, increasing the pressure can have a positive effect on increasing the solubility of both CO_2_ and N_2_ in the system, although this effect is more significant for CO_2_.

**Figure 10:**
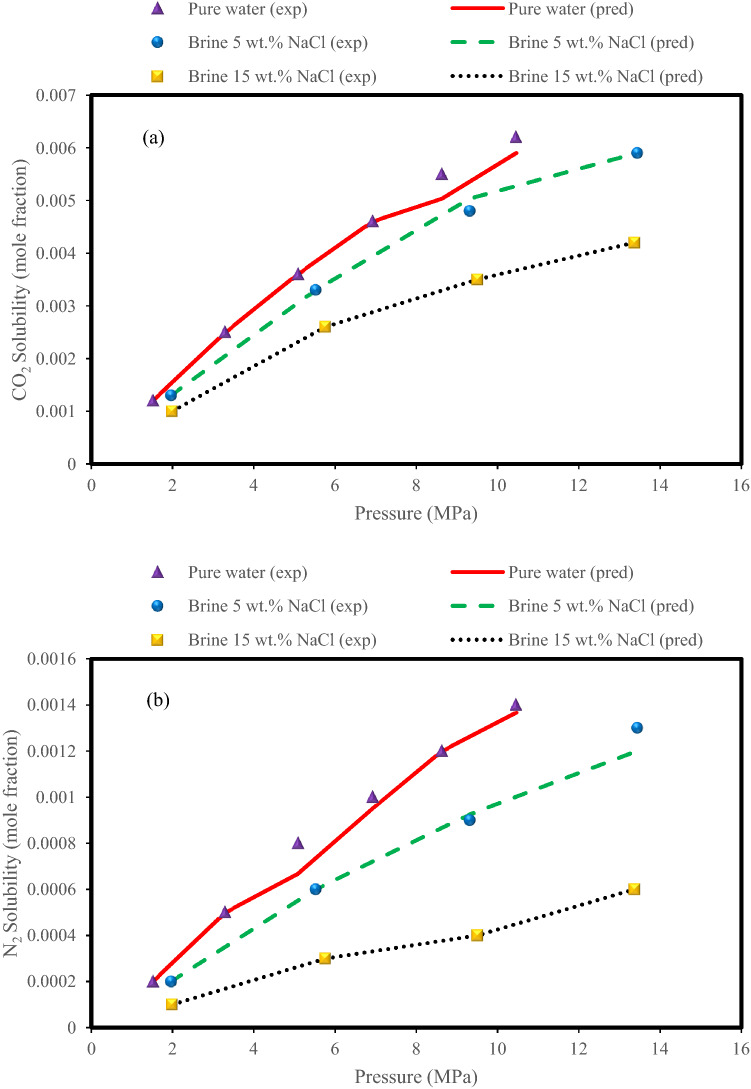
Effect of salinity on (**a**) CO_2_ solubility and (**b**) N_2_ solubility in the N_2_ (85.4%) + CO_2_ (14.6%) + H_2_O (brine) systems at a temperature of 273 K; experimental data^10^ with predictions of the Random Forest model.

Figure 10 shows the effect of pressure on the solubility of CO_2_ and N_2_ in aqueous systems with different salinity (pure water, 5% NaCl brine, and 15% NaCl brine). What can be seen in both Fig. 10a and b is the effect of salinity on system performance. For both CO_2_ and N_2_ gases, increasing the pressure increases the solubility, but it is noteworthy that increasing the salinity decreases the solubility of CO_2_ and N_2_. Therefore, increasing the concentration of NaCl in water, or in other words, an increase in the ionic strength of the solution, reduces the solubility of CO_2_ and N_2_. The salting-out phenomenon causes a reduction in CO_2_ and N_2_ solubility in water. The electrolytes influence water to dissolve less gas in this process. As salinity increases, more water molecules are attracted to the salt ions, reducing the amount of H^+^ and O_2_^−^ ions available to gather and separate the gas molecules, lowering CO_2_ and N_2_ solubility in the water^85^.

### Input parameters impact analysis

To study the influence of input parameters on the output of the model, a parameter called Relevancy factor was used. Relevancy factor is calculated as follows^86^:

17 $$r\left({inp}_{i},O\right)=\frac{\sum_{j=1}^{n}\left({inp}_{i,j}-{inp}_{m,i}\right)\left({O}_{j}-{O}_{m}\right)}{\sqrt{\sum_{j=1}^{n}{\left({inp}_{i,j}-{inp}_{m,i}\right)}^{2}\sum_{j=1}^{n}{\left({O}_{j}-{O}_{m}\right)}^{2}}}$$

Here, *inp*_*m,i*_, and *inp*_*i,j*_ indicate the average value, and the *j*th value of the *i*th input, respectively *O*_*j*_ refers to the *j*th value of predicted output, and *O*_*m*_ is the average of output data.

This parameter, which is between 1 and − 1, shows the effect of inputs on the output of the model as follows^87^:

- If the relevancy factor < 0, the impact of the input parameter on the output is decreasing. In other words, by increasing the desired parameter, the value of the output parameter decreases. On the other hand, the closer the relevancy factor to -1, the greater the influence.
- If the relevancy factor = 0, there is no relation between the input parameter and output or this relation is not monotonic.
- If the relevancy factor > 0, the impact of the input parameter on the output data is incremental. In other words, by increasing the desired parameter, the value of the output parameter also increases. Therefore, the closer the relevancy factor to 1, the greater the influence.

**Figure 11:**
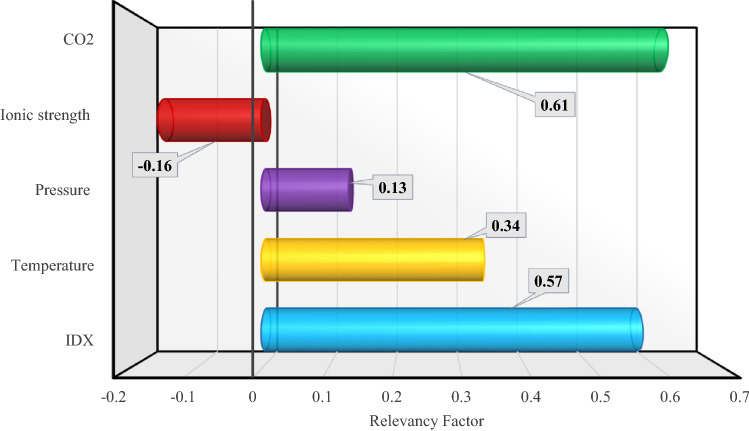
Evaluation of the input parameters' impact.

Figure 11 shows the relevancy factor value for the input parameters of the Random Forest model as the best model. According to this figure, the impact of temperature, pressure, and mole percent of CO_2_ in gaseous phase on the solubility of CO_2_–N_2_ mixture in aqueous solutions is increasing, and the impact of ionic strength is decreasing. Among the parameters whose relevancy factor values are positive, the mole percent of CO_2_ in gaseous phase with a relevancy factor of 0.61 has the most significant impact. Therefore, with increasing temperature, pressure, and the mole percent of CO_2_ in gaseous phase, the solubility of CO_2_–N_2_ mixture in water and brine solutions increases, and with increasing ionic strength, the solubility decreases.

### Implementation of Leverage method

The Leverage method^88–90^ was used to determine the applicability domain of the constructed Random Forest model and to identify any data that is suspect. The Leverage method, which is well-established analytically and visually through Williams' plot, is one of the most important approaches in outlier diagnosis. Standardized residuals (*R*), which reflect the differences of model’s outcomes from experimental observations, and Leverage values, which are the diagonal components of the hat matrix, are determined in this method. The following is the definition of the hat matrix^83^:

18 $$H=X{\left({X}^{T}X\right)}^{-1}{X}^{T}$$

here, *X*^*T*^ denotes the transpose of the matrix *X*, which is an (*m* $\times$ *n*) matrix, and *m* and *n* denote the number of data points and model input variables, respectively. In addition, the critical leverage (H*) is determined to be *3(n* + *1)/m*.

The proposed model's applicability domain is then graphically evaluated by displaying the standardized residuals versus leverage values. If most of the data points were located in the limits of $0\le H\le {H}^{*}$, and $-3\le R\le 3$, the created model is considered trustworthy and its estimations are made in the applicability domain^91^.

**Figure 12:**
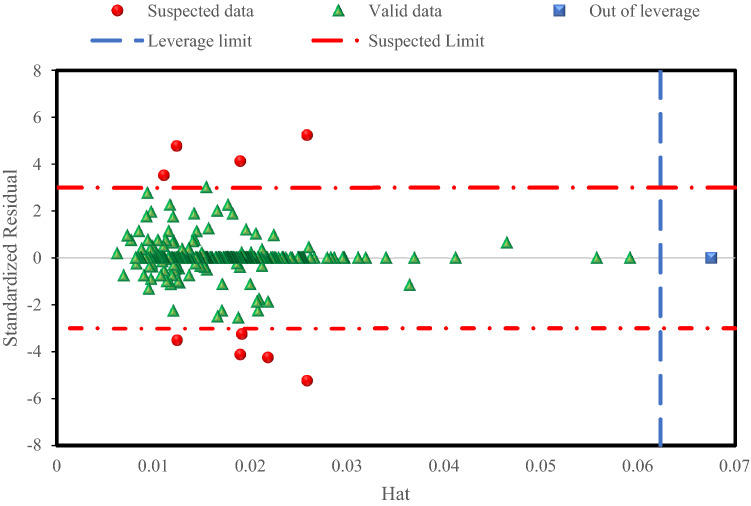
William's plot for the outlier detection using the Random Forest model.

Following that, as shown in Fig. 12, William's plot is utilized to determine the Random Forest model's applicable scope and outliers. As shown in Fig. 12, the majority of data falls between $0\le H\le 0.062$, and $-3\le R\le 3$, indicating that the experimental results are of excellent quality and the Random Forest model is quite reliable. Suspicious data are data points with *R* > 3 or *R* < − 3, linked with a high level of doubt. Out of Leverage data are data points with *H* > 0.062, and $-3\le R\le 3$ beyond the Random Forest model's applicability range. Only nine data points were identified to be as suspected data and one outlier exists in the solubility databank, which proves the high validity of the experimental databank used for modelling. Eight suspected data points along with one outlier belong to the training subset and one suspected data point belongs to the test subset, which is specified in the Supplementary file.

## Conclusions

In this study, using 289 laboratory data and six intelligent models including DT, GBDT, AdaBoost-DT, AdaBoost-SVR, GB-SVR, and Random Forest, the solubility of CO_2_ and N_2_ in the systems of CO_2_–N_2_ mixture and aqueous solutions was predicted and comparing their results with thermodynamic models such as SRK, PR, VPT, and PC-SAFT led to the following conclusions:

1. Among the presented models, the Random Forest model with an AAPRE value of 2.84% has the best results. GB-SVR and DT models then have the closest predictions with AAPRE values of 6.43% and 7.41%, respectively. After these models, AdaBoost-SVR, GB-DT, and AdaBoost-DT are ranked in terms of good predictions, respectively. Therefore, intelligent models are very efficient and reliable compared to equations of state.
2. Generally, the equations of state used in this work overestimate the solubility of CO_2_ in the aqueous system by increasing the pressure; however, this is the opposite for N_2_ except for the PR equation of state for all other models.
3. For solubility of CO_2_ present in gaseous mixtures in aqueous systems, the PC-SAFT model, and for solubility of N_2_, the VPT model had the best results among the equations of state.
4. Increasing the CO_2_ content in the gas mixture increases the solubility of CO_2_ in the system and, conversely, decreases the solubility of N_2_ at constant temperature and pressure.
5. Increasing the water salinity causes the reduction of CO_2_ and N_2_ solubility in water.
6. The impact of mole percent of CO_2_ in gaseous phase, temperature, and pressure on increasing the solubility of CO_2_ and N_2_ in water is incremental, and the impact of ionic strength on the solubility of CO_2_ and N_2_ in water is decreasing.

## Supplementary Information

Supplementary Information.

## Abbreviations

AAPRE: Average absolute percent relative error
SD: Standard deviation
SRK: Soave–Redlich–Kwong
SAFT: Statistical associating fluid theory
RMSE: Root mean square error
PR: Peng–Robinson
VPT: Valderrama–Patel–Teja
PC-SAFT: Perturbed-chain statistical associating fluid theory
EOS: Equation of state
GB: Gradient boosting
AdaBoost: Adaptive boosting
RF: Random forest
DT: Decision tree
SAFT: Statistical associating fluid theory
SVM: Support vector machine
SVR: Support vector machine for regression
SVC: Support vector machine for classification
